# Metagenomics to Detect and Characterize Viruses in Food Samples at Genome Level? Lessons Learnt from a Norovirus Study

**DOI:** 10.3390/foods11213348

**Published:** 2022-10-25

**Authors:** Florence E. Buytaers, Bavo Verhaegen, Mathieu Gand, Jolien D’aes, Kevin Vanneste, Nancy H. C. Roosens, Kathleen Marchal, Sarah Denayer, Sigrid C. J. De Keersmaecker

**Affiliations:** 1 Transversal Activities in Applied Genomics, Sciensano, 1050 Brussels, Belgium; 2 Department of Plant Biotechnology and Bioinformatics, Ghent University, 9052 Ghent, Belgium; 3 National Reference Laboratory (NRL) for Food-Borne Outbreaks and NRL Food-Borne Viruses, Food-Borne Pathogens, Sciensano, 1050 Brussels, Belgium; 4 Department of Information Technology, IDlab, IMEC, Ghent University, 9052 Ghent, Belgium

**Keywords:** metagenomics, norovirus, food, typing, Oxford Nanopore sequencing, adaptive sampling

## Abstract

In this proof-of-concept study on food contaminated with norovirus, we investigated the feasibility of metagenomics as a new method to obtain the whole genome sequence of the virus and perform strain level characterization but also relate to human cases in order to resolve foodborne outbreaks. We tested several preparation methods to determine if a more open sequencing approach, i.e., shotgun metagenomics, or a more targeted approach, including hybrid capture, was the most appropriate. The genetic material was sequenced using Oxford Nanopore technologies with or without adaptive sampling, and the data were analyzed with an in-house bioinformatics workflow. We showed that a viral genome sequence could be obtained for phylogenetic analysis with shotgun metagenomics if the contamination load was sufficiently high or after hybrid capture for lower contamination. Relatedness to human cases goes well beyond the results obtained with the current qPCR methods. This workflow was also tested on a publicly available dataset of food spiked with norovirus and hepatitis A virus. This allowed us to prove that we could detect even fewer genome copies and two viruses present in a sample using shotgun metagenomics. We share the lessons learnt on the satisfactory and unsatisfactory results in an attempt to advance the field.

## 1. Introduction

Foodborne viruses, in particular noroviruses, have been described as the contaminant causing the largest number of cases of foodborne diseases worldwide [1]. Norovirus was one of the most frequently reported causative agents for foodborne outbreaks in Europe in past years [2,3,4], with frozen soft fruits and shellfish named as important sources [5,6]. Norovirus is constituted of a positive RNA strand of about 7 thousand bases long. It is characterized based on dual typing of the ORF1 and ORF2 regions [7] into genogroups and genotypes. The genogroups GI and GII, along with GIV (less common), can be pathogenic in humans, while norovirus GV is harmful in murines. The minimal infectious dose for norovirus is approximately a thousand viral particles [5]. Viruses need host cells to replicate, making it more difficult to use culture-based detection methods for viral foodborne contaminants. Therefore, the conventional approach to detect and characterize these viruses in food is real-time polymerase chain reaction (qPCR) [8]. This method allows detection of specific genetic fragments of the pathogen after reverse transcription of the extracted RNA to complementary DNA (cDNA). If the result is positive, the test can be followed by a Sanger sequencing of specific regions of the genome to obtain the genotype of the virus [9]. However, detection of norovirus in food leftovers that might be linked to an outbreak has been reported as challenging because of the low contamination dose and the heterogeneity of the contamination [10,11,12], and even when the virus can be detected, it cannot always be further typed to be compared with the human cases [13]. Moreover, these conventional approaches do not deliver a full characterization of the complete genome of the virus, and therefore do not allow determining accurate relatedness of cases by performing phylogeny, which is often required in outbreak investigation. New outbreaks have been found to be caused by recombinant noroviruses, for which the sequencing of the overlapping region between ORF1 and ORF2 is necessary for a correct genotyping [7]. Finally, hepatitis A virus (HAV), another common foodborne virus [2], shares some similarities with norovirus but also the same contamination routes. Both RNA viruses can be tested simultaneously in a multiplex qPCR assay [7]. However, this is not done systematically, and other viruses or pathogens might be present in the food without being detected. Therefore, the conventional methods have their limitations for foodborne virus detection and characterization.

In recent years, metagenomics approaches, based on the sequencing of all genetic material of a sample, have been developed as an alternative method for various applications including the detection and characterization of viruses [14,15,16,17]. The study in food samples, with a commonly reported low contamination dose compared to clinical samples, would be particularly challenging. In 2018, Bartsch et al. studied frozen strawberry samples linked to a norovirus outbreak. Out of 29 million sequencing reads, only 2 could be matched to the sequences of norovirus from patients with high identity because mostly plant and bacterial material was sequenced [5]. Similarly, Yang et al. investigated norovirus and HAV that were artificially added at low concentrations in celery with a shotgun metagenomics approach [18]. They were able to infer the genotype of the spiked strains by mapping to a database of norovirus and HAV. However, a more open profiling approach did not succeed on their dataset and they did not obtain a genome to perform relatedness studies in case of outbreaks. To circumvent the low amount of viral reads, another previously documented approach is shotgun metagenomics sequencing after whole transcriptome amplification (WTA). Such a method was tested for the detection of viruses on various food matrices [19,20] but norovirus was not detected possibly due to an insufficient sequencing depth. Most of these studies have used short read sequencing. However, real-time long-read sequencing from Oxford Nanopore Technologies (ONT, using MinION and Flongle) might offer an interesting alternative for a lower price and turnaround time, and the possibility to sequence bigger fragments of the virus genome in one read. Furthermore, ONT has recently integrated a new mode called adaptive sampling to its GridION devices, allowing selective sequencing of DNA based on the similarity to reference sequences provided to the instrument [21]. This might offer targeted sequencing of the pathogen(s) present in the food matrix, without targeted RNA or cDNA preparation. For this purpose, the method would need a curated database of possible foodborne pathogens (bacteria, viruses, parasites, etc.), but this application has not been tested yet. Alternatively, some studies have focused on the enrichment in viral load before sequencing. This can be conducted before the RNA extraction protocol [22,23,24]. In particular, ultracentrifugation was performed to enrich for viral particles in the abovementioned study by Yang et al. [18]. Other studies have aimed to specifically increase the viral genetic material after the RNA extraction. One of the methods that have been previously presented is based on the removal of the background ribosomal RNA (rRNA). This was successful on clinical samples in which the human rRNA was depleted, and even allowed phylogenetic analysis of the detected strains [25,26]. It was also used for the detection of plant viruses after removal of the plant rRNA using FastSelect Plant and sequencing on Flongle flow cells [27]. An alternative method is to use beads to capture polyadenylated RNA, as norovirus and HAV present a poly(A) tail. This has been described to increase the norovirus loads in stool samples 40 times [28], but it has not yet been tested for the lower contamination dose in food samples. Finally, target enrichment of a virus of interest is possible using probes to capture the cDNA by hybridization, and washing away of the non-bound DNA. This has already been performed for norovirus in clinical but also sewage samples using probes developed specifically for human noroviruses proposed in the SureSelect products [29,30,31]. This method provided a high coverage of the norovirus genome in these samples; however, it has not yet been tested on food matrices. Moreover, very few studies have accompanied the hybrid capture with long-read sequencing [32].

The goal of this proof-of-concept study was to test several sample preparation and sequencing methods previously described, to determine which workflow could be used to detect and characterize foodborne viruses in food samples and allow us to obtain relatedness by phylogeny, taking norovirus in food matrices as a case study. Our study aims to serve as guidance for future work in the field. Therefore, we tested if we could obtain the genome of the virus with each method, because they had not all been used for low-contamination levels in food previously. We then performed a relatedness study with a phylogenetic tree when the viral genome was obtained, which was only previously performed in very few studies for viral food contamination. We present this work with the intention of providing a workflow that could later on, after thorough validation on a large set of samples, be implemented in routine settings. Therefore, we did not alter the validated RNA extraction protocol stated in ISO 15216-2 [8], currently used in the national reference laboratories (NRLs). Moreover, while most studies have been previously performed using Illumina sequencing, we decided to perform the sequencing on ONT flow cells and Flongles, because of the limited amount of samples received at the Belgian NRL for norovirus detection. A bioinformatics workflow was developed in-house in order to analyze the sequenced data obtained from different samples (different food matrices, different contamination loads) with each sample preparation or sequencing method of this proof of concept. It was also tested on a publicly available dataset of another food matrix (co-)spiked with norovirus and/or HAV at different contamination loads. The bioinformatics analysis included data quality checks and filtering, profiling to detect the presence of a virus in the sample without a priori knowledge, reference-based mapping, and building of a consensus sequence. This sequence was then typed and used for phylogenetic investigation. This relatedness characterization allows going well beyond the results obtained with the current methods of analysis of norovirus in food.

## 2. Materials and Methods

### 2.1. Samples

**Figure 1:**
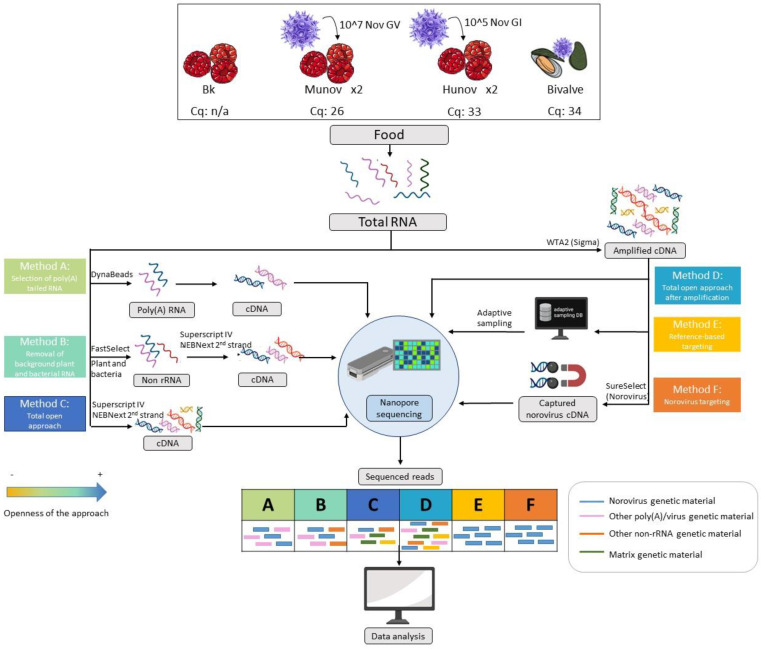
Overview of the different samples and methods of preparation of the genetic material tested in the study, starting from the total RNA extracted from the food sample following ISO 15216-2. Raspberries were spiked with norovirus GI at a concentration of 10^5^ genome copies per 25 g (hunov, Cq 33), norovirus GV at a concentration of 10^7^ (munov, Cq 26), or kept as a blank (Bk, not detected by qPCR). The spiking was repeated twice. A naturally contaminated bivalve sample (bivalve, Cq 34) was also investigated. Each method is explained in detail in Section 2 (Materials and Methods). Three methods were based on the total RNA (methods A-B-C) and three methods were based on the whole transcriptome amplification of the total RNA (methods D-E-F). The theoretical read outputs after sequencing are displayed for each method. The methods are classified per openness of the approach with a color code (orange least open, blue most open). The blue RNA/cDNA/reads represent the genetic material from norovirus in the sample while the other colors represent genetic materials from other origins, as indicated in the figure.

One kilogram of frozen raspberries was bought at a local store and was divided in parts of 25 g that were used for extraction (bk, Figure 1) or spiked with 5 lenticule discs of human norovirus GI.7 (Public Health England culture collection, Salisbury, UK, mean concentration of 1.9 × 10^4^ genome copies per lenticule disc), or with 100 µL of murine norovirus GV from the VIRSeek Murine Norovirus kit (Eurofins Genescan Technologies GmbH, Freiburg, Germany, mean concentration of 10^8^ genome copies per mL). This spiking led to a concentration of 10^5^ genome copies per 25 g for the norovirus GI (hunov, Figure 1) and 10^7^ genome copies per 25 g for the norovirus GV (munov, Figure 1). Two biological replicates of the spiking with the human norovirus (hunov1 and 2, Figure 1) and the murine norovirus (munov1 and 2, Figure 1) were performed on the same batch of raspberries.

A shellfish (i.e., bivalve) sample (bivalve, Figure 1) received at the Belgian NRL and naturally contaminated with norovirus (positive in qPCR with a Cq of 34 for the genogroup GII) was also included in the study.

### 2.2. RNA Extraction

For the blank and the spiked raspberries (bk, hunov, munov), ISO 15216-2 was followed with the recommendations for soft fruits, but without addition of mengovirus as process control during our method development, to increase the chance of sequencing norovirus genetic material. This protocol consists in several steps of shaking, incubation, centrifugation, and pH adjustment [8]. The final aqueous phase was used for RNA extraction.

For the bivalve sample, the sample preparation also followed ISO 15216-2 but for bivalves, and the addition of mengovirus as process control was also omitted. The sample preparation consisted in addition of proteinase K to 2 g of starting material, followed by centrifugation. The supernatant was used for RNA extraction.

The RNA extraction was conducted using the Nuclisens MiniMAG kit according to the manufacturer’s instructions (BioMérieux, Marcy-l’Étoile, France), which is the accredited procedure at the Belgian NRL. The final RNA of the raspberry samples was eluted in 100 µL of elution buffer. The RNA extraction of the spiked (and blank) raspberry samples was repeated 5 times and the eluates were pooled in order to have sufficient genetic material for subsequent tests (a total of 500 µL).

The presence of norovirus in the RNA pools was analyzed with qPCR (Figure 1, Table 1) using NovGI/GII @ceeramTools food kit multiplex (Biomérieux, Marcy-l’Etoile, France) to detect the human norovirus in accordance with the specifications of the ISO 15216-2, or using the VIRSeek Murine Norovirus kit (Eurofins Genescan Technologies GmbH, Freibourg, Germany) to detect the murine norovirus.

### 2.3. Genetic Material Preparation

Six sample preparation workflows were tested in this study (Figure 1).

#### 2.3.1. Method A: Poly(A) RNA Capture

The polyadenylated RNA was captured from the total RNA using DynaBeads mRNA DIRECT Purification Kit (Thermo Fisher Scientific, Waltham, MA, USA) following the protocol described by Fonager et al. [28]. The captured and eluted RNA was tested for the presence of norovirus with the same qPCR as described for the total RNA, and then reverse transcribed using SuperScript IV (Invitrogen, Thermo Fisher Scientific, Waltham, MA, USA) with random hexamers following the manufacturer’s instructions for the first strand. The second strand was then synthetized using the NEBNext Ultra non-directional RNA second strand synthesis module (New England Biolabs, Ipswich, MA, USA) following the manufacturer’s instructions. The prepared cDNA was cleaned using AMPure beads (Beckman Coulter, Brea, CA, USA) at a ratio of 1:1 and two rounds of washing with 200 µL 70% ethanol were conducted. It was then eluted in nuclease-free water in the same volume as the starting volume after leaving at room temperature for 2 min.

#### 2.3.2. Method B: Plant and Bacteria rRNA Depletion

Plant and bacterial ribosomal RNA was depleted from the total RNA using the FastSelect rRNA Plant and 5S/16S/23S kits (Qiagen, Hilden, Germany). The protocol of the FastSelect 5S/16S/23S was followed with addition of 1 µL of FastSelect Plant to the mix and no fragmentation The reverse transcription of the two strands of cDNA was then conducted on the non-rRNA as described in method A. The prepared cDNA was cleaned using AMPure beads as for method A. The cDNA was then tested for the presence of human or murine norovirus with qPCR as described for the total RNA.

#### 2.3.3. Method C: Shotgun cDNA

The first-strand cDNA synthesis was performed on the pooled total RNA using SuperScript IV and the second strand of cDNA was synthetized using the NEBNext Ultra non-directional RNA second strand synthesis module as described for method A. The prepared cDNA was then cleaned using AMPure beads similarly as for method A.

#### 2.3.4. Methods D and E: Shotgun-Amplified cDNA

The pooled total RNA was reverse transcribed and amplified using the whole transcriptome amplification 2 kit (Sigma-Aldrich, Saint-Louis, MO, USA) according to the manufacturer’s instructions. The prepared cDNA was cleaned using AMPure beads in the same way as for method A. The choice of the amplification kit has previously been shown to have an impact when sequencing long reads [33], and the WTA2 from Sigma-Aldrich was recommended over other products to avoid the sequencing of chimeric junctions that can be created during a ligation step.

#### 2.3.5. Method F: Amplified Norovirus Captured cDNA

After amplification and bead cleaning as described in method D, the amplified cDNA was sheared to a size of about 1 kb using the covaris microTUBE AFA Fiber pre-slit snap-cap 6 × 16 mm PN 520,045 with the insert microTUBE 130µL, with peak incident power 50, duty factor 2%, 200 cycles per burst for 30 s (Covaris, Woburn, MA, USA). Although not ideal for long-read sequencing, the cDNA shearing was recommended for the hybridization. SureSelect XT2 was then used to capture the norovirus sheared cDNA with the PanNoro panel of probes (Agilent, Santa Clara, CA, USA) according to the manufacturer’s instructions, including recommendations for long-read sequencing. The amplification was necessary to have sufficient starting cDNA material for the SureSelect protocol.

### 2.4. Long Read Sequencing

#### 2.4.1. Library Preparation

All samples to sequence were prepared using the Ligation sequencing kit for genomic DNA (SQK-LSK109; Oxford Nanopore Technologies, Oxford, UK) according to the manufacturer’s recommendations for the specific flow cell used for sequencing. When the recommended input amount of genetic material was not met, the maximum volume of starting material (48 µL) was used.

Several sequencing methods were tested in this study: sequencing on MinION flow cells, without or with adaptive sampling (see method E), and sequencing on Flongle flow cells.

#### 2.4.2. MinION Sequencing

The prepared libraries (except for method E) were loaded on one Spot-ON MinION flow cell (FLO-MIN 106D; R9.4.1 version) per sample, and a 72 h sequencing run was started on a Mk1C or a GridION device.

#### 2.4.3. Method E: Adaptive Sampling

The prepared libraries of shotgun-amplified cDNA (prepared following the same protocol as described for method D) were loaded on one MinION flow cell (R9.4.1) per sample on a GridION device. A 72 h sequencing run was started using the adaptive sampling option of the MinKNOW software version 22.08.9 (focal), with a FASTA file of norovirus and hepatitis A reference genomes from NCBI (listed in Supplementary Materials File S1). The number of reads, median read length, median read quality, and read length N50 for each sample are presented in Figure 1. All reads were analyzed as such (“all reads”) or only the reads characterized as “stop_receiving” from the adaptive sampling csv file were analyzed separately. The “stop receiving” reads correspond only to reads that matched the database, and not the reads that had started to be sequenced before a decision was made by the instrument about their resemblance to the database.

#### 2.4.4. Flongle Sequencing

The libraries prepared with adjusted volumes for Flongle sequencing were loaded on one Flongle flow cell FLO-FLG001 (R9.4.1 version) per sample, and a 24 h sequencing run was started.

### 2.5. ONT Data Analysis

**Figure 2:**
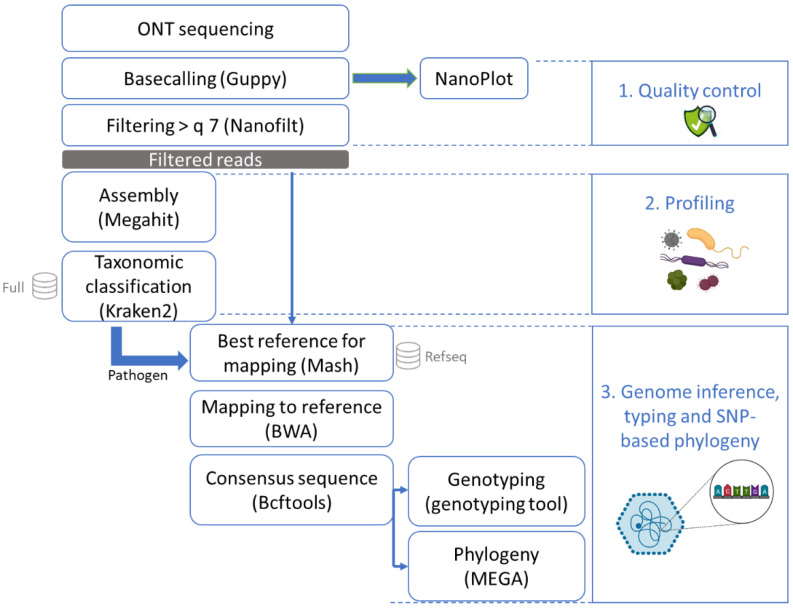
Bioinformatics workflow after sequencing with ONT (MinION or Flongle). After sequencing, the data were basecalled and checked for quality control. The reads were then all assembled and the contigs were put through a taxonomic classification tool with a database of mammals, archaea, bacteria, fungi, human, protozoa, and viruses. Once a pathogen was recognized in this profiling step, all reads were mapped to the RefSeq database using Mash to obtain the best matching reference genome. This reference genome was used to perform mapping and to build a consensus sequence which could then be typed and used for phylogenetic analysis.

The data analysis of the ONT sequenced reads (Figure 2) started with basecalling using Guppy version 5.0.7 (Oxford Nanopore Technologies) in super high-accuracy mode on a GPU server. Statistics were obtained from the basecalled reads using NanoPlot version 1.36.2 [34] for quality assessment (Table 1). The reads were further filtered to retain only those with a quality higher than 7 using NanoFilt version 2.8.0 [34].

The high-quality reads were then assembled with Megahit version 1.1.3 [35] using as k-list 21, 33, 55, 77, 99, 127, 155, 183, 211, 239. This tool was selected due to the relatively low obtained read lengths that were not compatible with most assemblers designed for long reads. The k-list was designed to cover the diversity of read lengths that we obtained (see Table 1). Taxonomic classification was conducted on the contigs using Kraken2 version 2.1.1 [36] with default parameters and an in-house database containing all NCBI RefSeq genome entries with the “complete genome” assembly level (database accessed 11 February 2021; [37]) accession prefixes NC, NW, AC, NG, NT, NS, and NZ of the following taxonomic groups: archaea, bacteria, fungi, human, protozoa, and viruses. To determine if a foodborne pathogen was present in the sample, manual inspection of the Kraken2 results was performed. Here, we only report the number of contigs that corresponded to norovirus. The profiling was conducted on the contigs obtained with Megahit instead of the reads in order to increase the trust in the result of the presence of species at very low concentration, such as the low spiked concentration of the pathogenic virus of interest [38].

All high-quality reads were then entered into Mash version 2.2 [39] to estimate the distance compared to the entire RefSeq database (refseq.genomes.k21s1000.msh) using mash screen with standard parameters. The results were sorted, and the best hit corresponding to the pathogen detected with Kraken2 (norovirus or hepatitis A) was used for reference-based assembly. The reads were mapped to the reference using BWA-MEM version 0.7.17 [40] with the ont2d parameter. Variant calling was then performed using Bcftools version 1.9 [41]. Bcftools mpileup was used with the parameters –A –B –q 0 and –Q 0. Bcftools call was used to output variants sites only, with the ploidy parameter set as 1. The creation of the consensus sequence was executed with bcftools consensus using the reference previously selected with Mash. Samtools version 1.9 [41] was used to calculate the read depth. Subsequently, the norovirus consensus sequence was typed using the online Norovirus Typing Tool version 2.0 (rivm.nl/mpf/typingtool/norovirus/ [42]). Finally, after multiple sequence alignment using ClustalW on MEGA-X [43] with the default parameters, a maximum likelihood phylogenetic tree was constructed based on the consensus sequences as well as norovirus sequences from NCBI [44] on MEGA-X with 100 bootstraps, using the Tamura–Nei model with partial deletion and other default parameters.

### 2.6. Adaptation of the Bioinformatics Workflow to the Analysis of Illumina Data and the Detection and Characterization of HAV

In order to test our method on samples in which two viruses were present, but also with a spiking at a lower concentration, we used the publicly available data from 13 samples sequenced on Illumina MiSeq from Yang et al. [18], downloaded from the NCBI Sequence Read archive under BioProject PRJNA377525. Briefly, Yang et al. spiked celery with norovirus GII at various concentrations (i.e., 10^3^ to 10^5^ viral RNA copies in 50 g) and co-spiked two strains of norovirus GII (GII.4 and GII.6) or one strain of norovirus GII and one strain of hepatitis A virus (HAV HM175/18f.1, genotype IB). The RNA was extracted using a QIAamp Viral RNA kit (Qiagen, Hilden, Germany) after ultracentrifugation-based viral particle enrichment. The obtained RNA was reverse transcribed and sequenced on an Illumina MiSeq generating paired-end 100 bp reads [18]. It was analyzed with the same data analysis workflow, with some modifications due to the difference in sequencing technology and the reads being paired: basecalling and quality filtering were replaced by trimming using Trimmomatic version 0.38 [45] on paired-end reads using the truSeq3 adapter sequence, the assembly was performed with megahit with the conventional k-list (21, 29, 39, 59, 79, 99, 119, 141), and BWA mem was used without the ont2d parameter. The online Hepatitis A Virus Genotyping tool version 1.0 (https://www.rivm.nl/mpf/typingtool/hav/ [42]) was used to type the consensus sequences obtained for the hepatitis A virus in the samples.

## 3. Results

In this study, we spiked raspberries with two genogroups of norovirus (norovirus GI at 10^5^ genome copies in 25 g of raspberries, hunov, and norovirus GV at 10^7^ genome copies in 25 g of raspberries, munov) to represent two contamination levels, of qPCR Cqs of respectively 33 and 26. The blank raspberries were also investigated as well as a bivalve sample naturally contaminated with norovirus GII with a qPCR Cq of 34 (lower contamination level than the spiked samples). Aiming specifically to present a method that could be later applicable in routine settings, we decided to follow the RNA extraction method used at the Belgian NRL, covered in ISO 15216-2. As we did not alter the RNA extraction method, we tested several post-extraction methods for genetic material preparation (Figure 1) in order to increase the presence of the virus of interest: i.e., poly(A) RNA capture (method A), plant and bacterial rRNA removal (method B), norovirus cDNA hybrid capture (method F) and whole transcriptome amplification (method D). A shotgun metagenomics protocol without specific treatment of the extract (method C) was also evaluated on the same spiked samples. All these prepared genetic materials were then sequenced using the ONT platform. Adaptive sampling during nanopore sequencing was also assessed for this case study as an alternative to the targeting of the virus with laboratory methods (method E). The main differences in input and output and how much they target norovirus are presented in Figure 1.

### 3.1. Selection at RNA Level: Subset of Poly(A) RNA (Method A) or Non-rRNA (Method B)

In order to decrease the complexity of the sample, a selection at RNA level was attempted with two different methods: capture of polyadenylated RNA (method A, Figure 1) and depletion of ribosomal RNA from plants and bacteria (method B, Figure 1). Both methods were tested on raspberry samples spiked at different contamination levels using human or murine norovirus. The resulting RNA was then tested via qPCR before reverse transcription. However, for both methods, norovirus was not detected by qPCR (data not shown) while it was detected in the total RNA prior to the sample treatment. These samples were therefore not used further for sequencing.

### 3.2. An Open Approach: Shotgun Sequencing of cDNA (Method C)

As the most straightforward and open approach, we tested the shotgun metagenomics sequencing of the samples after reverse transcription of the RNA (method C, Figure 1). This was performed on biological replicates of munov and hunov (Figure 1). After sequencing, tens of thousands to more than a million reads were produced for the spiked samples (Table 1) with a median read length of a few hundred base pairs and a quality of 9 to 10.

The sequenced reads were then analyzed through the developed bioinformatics workflow (Figure 2). As our previously published workflow [46] for the strain-level analysis of long-read sequencing data was not successful on the case study of this viral contamination (data not shown), we designed an alternative data analysis workflow (Figure 2). After assembly of the reads, a taxonomic classification was performed on the contigs. Norovirus was detected in contigs of the cDNA sequencing of the two munov samples (Table 2) and in one of the samples spiked with human norovirus (hunov, Table 2). The sequencing of the blank (non-spiked) raspberries led to the lowest amount of reads (bk, Table 1), of which none could be related to norovirus or another foodborne virus (Table 2). The high amounts of unclassified contigs probably corresponded to the raspberry genetic material, of which no reference was present in the database used for profiling.

The closest norovirus reference was then estimated from all reads, and this reference was used for mapping and generation of a consensus sequence, which was then typed (Table 2).

For munov, NC_008311.1 was the most similar reference in the RefSeq database. Thirty-five and one hundred and fifty-five reads mapped to this reference for the first and second replicate of the spiking, covering 85% and 99% of the norovirus genome, respectively (Table 2). Consensus sequences of 6304 and 7280 bases were obtained and could be correctly typed as norovirus GV (murine norovirus).

For the first replicate of hunov, NC_031324.1 was determined as the closest reference. Eighty-six reads covered 16 of the reference genome, and a consensus sequence of 1221 bases was obtained and correctly characterized as norovirus GI. The characterization went further to describe the strain as a norovirus GI.3P3, although the lenticule that was used to spike the sample was notified as norovirus GI.7 by the supplier.

**Figure 3:**
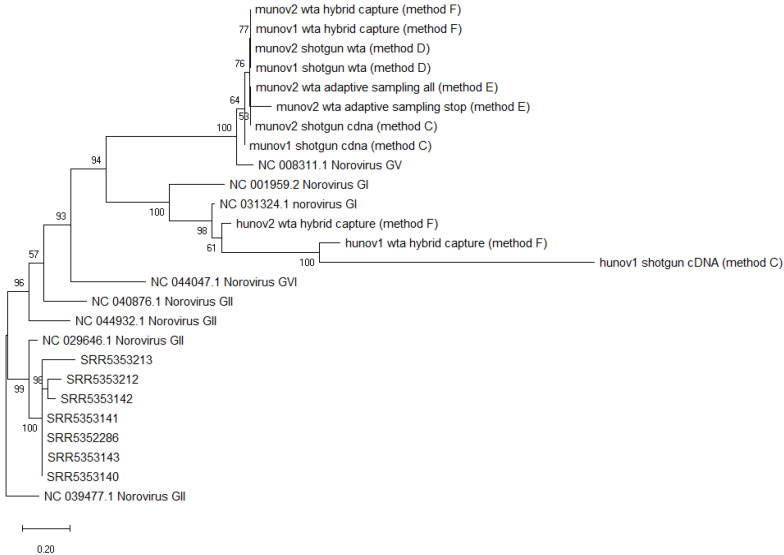
Phylogenetic tree of consensus sequences of all norovirus strains from metagenomics datasets and several reference strains as background. SRR535XXX: norovirus strains obtained from metagenomics samples of spiked celery [18]. Munov: in biological duplicates (1,2), norovirus strain obtained from raspberries spiked with murine norovirus GV. Hunov: in biological duplicates (1,2), norovirus strain obtained from raspberries spiked with human norovirus GI. Shotgun cDNA: complementary DNA after reverse transcription (method C). Shotgun wta: cDNA after whole transcriptome amplification (Method D). Wta hybrid capture: cDNA after whole transcriptome amplification with target enrichment for norovirus using SureSelect (Method F). Wta adaptive sampling: cDNA after whole transcriptome amplification, sequenced with adaptive sampling (Method E). All: all reads sequenced. Stop: only “stop receiving” reads after adaptive sampling of whole transcriptome amplification cDNA. Background strains obtained from NCBI [44]. The scale represents the distance of 20% genetic variation.

The consensus sequences obtained for the two munov and hunov replicates with the different sample preparation methods were placed in a phylogenetic tree, and clustered together respectively, while separating from other norovirus GV (murine) or norovirus GI (human) reference genomes (Figure 3).

### 3.3. An Open Approach with Increased Input Genetic Material: Shotgun Sequencing of Amplified cDNA (Method D)

In order to increase the overall sequencing output, including the virus sequenced reads, the RNA was reverse transcribed with an amplification kit. All the amplified cDNA was then sequenced. This was done on the two biological replicates of munov and hunov, but also on the bivalve sample. The blank raspberries (bk) were also sequenced after the same amplification.

After amplification, the number of reads sequenced respectively for each sample was increased by 10 to 100 times (Table 1). The read quality increased as well, but the read length and N50 slightly decreased.

After assembly, norovirus could only be detected in the contigs of the munov samples, not in hunov, bk nor the bivalve sample (Table 2). NC_008311.1 was again identified as the most similar reference in the database (Table 2). There were 743 and 1312 reads mapped to this reference, covering 91% and 98% of the genome for the first and second spiking experiments, respectively. Consensus sequences of 6694 and 7271 bases were obtained and correctly characterized as norovirus GV (murine norovirus).

The consensus sequences of the two norovirus strains from the munov samples could be placed correctly in a phylogenetic tree, clustering with other murine norovirus (GV) strains but separated from other non-GV norovirus genomes (Figure 3).

### 3.4. Capturing Norovirus in the Amplified Genetic Material: Sequencing Amplified Targeted cDNA (Method F)

In order to increase the viral load in the cDNA, the norovirus genome can be targeted with a hybridization and capture method (SureSelect) based on a panel of probes designed based on human strains of norovirus. In order to have sufficient starting material for this protocol, the extracted RNA was first amplified as in method D. This was performed on the two biological replicates of munov and hunov, but also on the bivalve sample (Figure 1). This double amplification (i.e., whole transcriptome amplification and capture of norovirus cDNA) led to the highest number of sequenced reads, with a quality above 12 but a short length of approximatively 400 bp (Table 1), while the shearing performed for this protocol was expected to create fragments of 1 kb.

After assembly of the reads, norovirus was detected in the contigs of munov1 and 2 and hunov1 and 2, but not in the bivalve sample.

The closest reference to the munov samples was NC_008311.1 as obtained with the other methods (Table 2): 98% and 100% of its genome was covered, respectively, with 2786 and 18,030 reads mapping to the reference. A consensus sequence of 7255 and 7381 bases was obtained and correctly characterized as norovirus GV (murine norovirus).

For the hunov samples, NC_031324.1 was the closest reference; 40% and 78% of the genome was covered, respectively, with 301 and 2636 reads. A consensus sequence of 3115 and 6071 bases was obtained, and it was correctly typed as norovirus GI.

The obtained consensus sequences were placed in a phylogenetic tree (Figure 3). The two norovirus strains from the munov samples clustered with the other murine norovirus strains while the two norovirus strains from the hunov samples clustered with the other human norovirus strains. Both clusters separated from genomes belonging to other genogroups.

### 3.5. Selection of Viral Genetic Material during the Sequencing through the Pore: Adaptive Sampling of the Amplified cDNA (Method E)

As an alternative method to the capture of the virus with the SureSelect kit (method F), the DNA fragments corresponding to the norovirus were selected during the sequencing using adaptive sampling. This method compares the read being sequenced in real time to a database (in this case, a database of norovirus and hepatitis A virus sequences, Supplementary Materials File S1). If the read differs from the sequences in the database, the pore releases the cDNA strand and captures another cDNA to sequence. This way, the targeted species are preferentially sequenced and should be represented in higher proportions in the reads. This was performed on the second biological replicate of hunov and munov.

The complete set of sequenced reads was analyzed, but we also analyzed separately the reads characterized as “stop receiving”, which only represented the reads that matched to the reference genomes in the database. One hundred and sixty-one reads were tagged as “stop receiving” for munov2, while none were tagged as such for hunov2 (Table 2). After assembly, norovirus could be detected by taxonomic classification only in the munov2 sample for all the reads or the stop receiving reads (Table 2). Mash determined NC_008311.1 as the closest norovirus reference, and 91% of its genome was covered by all the sequenced reads (compared to 98% for the same amplified DNA sample without adaptive sampling). A consensus sequence of 6978 bases could be constructed based on the 711 reads that mapped to the reference (compared to 1312 for the same sample without adaptive sampling), and it was typed as norovirus GV. For the stop receiving reads, only 61% of the reference genome could be covered and all the 161 reads mapped to the reference. A consensus sequence of 4474 bases was constructed and was characterized as norovirus GV.

The consensus sequence of murine norovirus obtained from all reads, or just the “stop receiving” reads, could be placed in a phylogenetic tree, and clustered with other murine noroviruses from the study, separated from another norovirus GV and from noroviruses from other genogroups (Figure 3).

### 3.6. Flongle Sequencing as a Low-Output, Less Expensive Sequencing Alternative

Several samples were also sequenced on Flongle flow cells, in order to verify at which level of contamination a low-output, less expensive sequencing alternative would be able to detect and characterize the norovirus in the samples. The number of reads sequenced on Flongles was lower than expected (a Flongle should have about 10% of pores compared to a normal flow cell and therefore we expected 10% of output) (Table 1). In particular, the sample of amplified and captured cDNA (method F) only presented 12 reads (Table 1). For that sample, no further analysis was performed. The three other samples presented a few thousand sequenced reads (Table 1). After assembly, no contig could be recognized as norovirus by taxonomic classification (Table 2).

### 3.7. Assessing Our Bioinformatics Workflow for the Analysis of a Dataset Containing a Co-Spike of Norovirus and Hepatitis A Virus

In order to test the performance of our bioinformatics pipeline as an open approach method, we analyzed a publicly available dataset containing co-spikes of norovirus and hepatitis A virus. In 2017, Yang et al. spiked celery with norovirus GII at various concentrations and co-spiked two strains of norovirus GII or one strain of norovirus GII and one strain of hepatitis A virus. The RNA extracted from these samples was reverse transcribed and sequenced on an Illumina MiSeq [18]. We conducted the data analysis on their data with the workflow we developed, adapted to the investigation of reads from Illumina.

Our results (Table 3) show that norovirus was detected after taxonomic classification in 11/13 samples spiked with norovirus. Hepatitis A virus was detected in 4/4 samples spiked with hepatitis A virus (co-spiked with norovirus GII).

Mash picked the closest norovirus reference as NC_029646.1 in the 11/13 samples for which norovirus was previously detected in the profiling step. A consensus sequence was obtained for 7 samples, covering 23% to 56% of the reference genome, and correctly typed as norovirus GII or GII.P4. The consensus sequence was not obtained for the 2 samples co-spiked with 2 viral species for which there was no norovirus hit with mash (SRR5353214 and SRR5353215), and for the 4 samples co-spiked with 2 strains of norovirus GII, for which only 1 hit was obtained with mash and the 2 strains could not be resolved (SRR5353144, SRR5353145, SRR5353158, SRR5353159). Norovirus was detected in the 2/4 samples co-spiked with the 2 viral species spiked at a higher concentration of norovirus (10^6^ genome copies in 50 g of celery). The two norovirus consensus sequences obtained covered 23% and 41% of the reference genome and were correctly characterized as norovirus GI.P4. At the time of their study, Yang et al. were able to detect the norovirus GII.4 in all four samples co-spiked with HAV and norovirus, but they were not following a fully open approach as they were using a curated database of norovirus and HAV.

For hepatitis A virus, NC_001489.1 was the closest reference in our database for the four samples, and a consensus sequence covering more than 99% of the reference was obtained. It was correctly characterized as HAV I.B in all cases.

All obtained consensus sequences were then placed in a phylogenetic tree. All norovirus strains obtained from the samples of Yang et al. clustered together, while separating from other norovirus GII genomes (Figure 3). This was the expected result, as consensus sequences were obtained from samples all spiked with the same strain of norovirus GII.4.

## 4. Discussion

Foodborne viruses, and in particular norovirus, represent a major worldwide burden on our food safety. However, due to their very low contamination dose in food, they are particularly hard to detect in food products suspected to cause a foodborne outbreak. Moreover, the current methods to detect norovirus in food samples do not give the full information about its genome, nor allow relatedness analysis. In this study, we investigated metagenomics as a new alternative approach that would allow us to obtain the genome of the viral pathogen present in the food sample and perform relatedness analysis with phylogenetic trees. For all samples, we performed RNA extraction according to the ISO norm currently in practice at the Belgian NRL, in order to present a protocol that could be easy to implement as an alternative for these laboratories after formal validation of the full workflow. Because metagenomics enables sequencing all genetic material in the sample, only a few reads might belong to the virus of interest. We therefore tested different sample preparation and sequencing approaches with various degrees of targeting of the virus. We decided to sequence with the MinION or Flongle flow cells from ONT because they offer fast results (real-time sequencing) compared to, e.g., Illumina sequencing, but also because they are more cost-effective when only a few samples have to be sequenced at a time [46]. This would all help in a further application of the protocol in routine settings. In order to compare the results obtained for each method, we developed a bioinformatics workflow to analyze the data without a priori knowledge, profiling for the pathogen in the sample, obtaining its genome, characterizing it, and relating it to other sequences. This was done as a proof-of-concept to deliver the most suited protocol to investigate further for future implementation.

As the most open approach, we tried shotgun sequencing on the extracted RNA. We compared the results obtained with either reverse transcription (method C) or with whole transcriptome amplification (reverse transcription followed by random amplification, method D) in order to improve the input RNA amount. Amplification has been described in several studies as a necessary step for the detection of viruses with shotgun metagenomics [22,47], and ONT sequencing in particular requires high levels of starting genetic material. In the current study, amplification of the genetic material enhanced results for the detection and characterization of norovirus in the sample spiked with murine norovirus (GV) at a concentration of 10^7^ (longer consensus sequence and 10-fold increase in number of reads mapping to the reference) but not in the samples spiked at a lower concentration with norovirus GI and in the naturally contaminated bivalve sample. Therefore, although improving the result at high contamination level, unspecific amplification did not allow strain-level characterization and phylogeny at a lower contamination. Both with and without amplification, when the virus was detected during the profiling step, a consensus sequence could be obtained and it was typed and correctly placed in a phylogenetic tree. In one sample spiked with the human norovirus, the typing to genotype level was incorrect (based on the information we received from the supplier of the norovirus reference material); however, the genogroup was correctly determined for all samples. Moreover, phylogeny allows a relatedness at a higher discriminatory level than genotyping.

In order to increase the amount of sequenced reads corresponding to the pathogen and possibly decrease the host DNA sequenced (the food), we tested more targeted approaches. Because we wanted our workflow to be applicable in a routine laboratory, we decided to keep the conventional RNA extraction workflow described in ISO 15216-2. We then tested two post-RNA extraction methods to increase the norovirus load in the samples to be sequenced. We first tested an approach that could capture the polyadenylated RNA (method A, norovirus RNA harboring a poly(A) tail) and an approach depleting the ribosomal RNA from plants and bacteria (method B). Unfortunately, these two methods did not give the expected result, as norovirus could not be detected for any of our samples within the detection limit of the qPCR after following these two protocols. The explanation for this lack of success was probably the very low contamination load of norovirus in our samples. For the first method, the few RNA fragments belonging to the virus were probably lost during the poly(A) capture and washing step. A previous study had reported very good results with this method, but it was conducted on stool samples, with a much higher dose of the virus [28]. In the case of the ribosomal RNA depletion, the cause of our lack of success was possibly also a loss of norovirus RNA due to dilution during the protocol or washing out. Moreover, we observed that raspberry was not part of the sequences used to design this plant RNA depletion kit. Indeed, although this kit gave good results in other studies, it is not universal for all plants. In fact, a study of plant viruses using the same method followed by Flongle sequencing reported lower results when analyzing strawberries than peas [27]. Moreover, FastSelect does not exist for other eukaryotes (like bivalves) and therefore this method could not be applicable in a routine laboratory setting handling various types of food matrices.

Aiming to further improve the results, two other methods were tested that targeted directly the virus of interest, at the cDNA level, after amplification: a target enrichment using SureSelect based on capture using a panel of probes designed for human norovirus (method F), and an adaptive sampling during the nanopore sequencing based on a database containing references of norovirus (method E). The amplification was necessary for both methods in order to have sufficient input genetic material for the protocols. Moreover, although a protocol adapted for ONT sequencing was available for the SureSelect (received from the R&D team of Agilent), this method primarily aims at preparing a library for short reads sequencing and the cDNA had to be fragmented to an average size of 1 kb, which is not ideal for subsequent long-read sequencing. Our results showed that this double amplification was the only method able to detect and characterize the norovirus at both levels of artificial contamination (10^5^ and 10^7^ genome copies per 25 g of fruit). The obtained consensus sequence of the virus could be typed and correctly placed in a phylogenetic tree. This sample preparation method was, however, not able to lead to the detection of norovirus at a lower concentration in the naturally contaminated bivalve sample. SureSelect was indeed previously shown to work better with genome copy inputs higher than 10^4^ in previous tests from the company [48]. Although this is unfortunate, as the contamination load in food samples can be very low, this is in agreement with the results we obtained, as we could not detect norovirus after SureSelect enrichment in the sample with the lowest contamination (the bivalve sample). Yet, this approach is very selective as it can only target norovirus and no other viral pathogen in the sample, and it is based on a panel of probes, which might not recognize a novel variant. For these reasons, a new method associated with ONT sequencing was tested: adaptive sampling. We tested this approach with a database of noroviruses and hepatitis A viruses in order to be more open than the SureSelect approach targeting only noroviruses. Ideally, for our application, a database of references of all food pathogens should be provided to the software. In our case, we could see that this method did not improve the results compared to those obtained on the same genetic material without adaptive sampling. This is probably explained by the shortness of our cDNA fragments, as at least 400 bp have to pass through the pore for the software to determine if the DNA strand resembles the reference(s) [49], but our mean read length was close to 400 bp. For the sample spiked with human norovirus, no read was recognized as norovirus while for the sample spiked with murine norovirus, 161 reads were tagged as “stop receiving” during adaptive sampling, which means they corresponded to a reference in our database and the sequencing continued for this DNA fragment. This still represented a loss compared to the 711 reads that mapped to the reference when using all reads sequenced in the run. This could be improved by producing longer cDNA fragments to sequence or if the number of bases necessary for the tool to make its decision decreased in further updates from ONT. Consequently, and unfortunately, adaptive sampling was not a usable alternative for this case study at the time the experiments were conducted.

As our method was able to obtain results after MinION sequencing of several samples, the Flongle was tested as a less expensive alternative sequencing approach. Although we had an acceptable amount of active pores for Flongle sequencing, very few reads were obtained compared to with the MinION sequencing (less than the 1/10th that would be expected from the difference in number of pores), and norovirus could not be detected after data analysis in any of our samples. Flongle sequencing had not been used before on such low contamination loads in food. However, it had been described for the detection of food viruses in plants for routine use [27], but without indication of the contamination load. We believe that the contamination load in our samples was too low for Flongle technology to obtain sufficient reads. It has been acknowledged by ONT as an instrument that does not yet perform as efficiently as the MinION technology (already available for a longer time) and has pores that are more sensitive towards potential artefacts (e.g., the use of glass vials instead of plastic vials is required so as not to impact the sequencing). Therefore, a full characterization of the genome of the virus in contaminated food samples with Flongle sequencing is too challenging. However, Flongles might be optimized by the manufacturer in the future and could then be used for more complex cases.

As a final test of our initially envisaged open approach, we wanted to analyze food samples contaminated with another virus, e.g., hepatitis A virus. We worked with a previously published dataset of celery spiked with norovirus and hepatitis A virus [18]. This dataset was produced with an improved RNA extraction aiming at increasing the viral load in the extract by using ultracentrifugation and a commercial viral RNA extraction kit. Moreover, it was sequenced on an Illumina MiSeq. At the time of the publication, the authors were able to detect the viruses present in all the samples, even when two strains of norovirus were spiked in the same sample. After analysis with our bioinformatics workflow (revised for Illumina reads), we could detect norovirus in 11 out of 13 samples spiked with norovirus, and HAV in 4 out of 4 samples spiked with it, with a completely open approach. We then built a consensus sequence for these strains that could further be typed, but also placed in a phylogenetic tree. This goes beyond the analysis previously conducted on this dataset, and also the results that can be obtained with the currently available conventional methods. Notably, at the time of the publication of these sequences, a very open profiling method, Kraken, did not detect the viruses, as reported by the authors. Five years later, we could detect these viruses with the same tool, probably due to the update in the databases within this time period and the assembly of the reads prior to the taxonomic classification. Our analysis was able to detect two different viral species when co-spiked in the same celery sample, which could then be characterized to the genotype level. Because our analysis workflow was based on the best hit with Mash, we could, however, not separate two strains of the same genogroup, and the database used (Refseq) only contained one reference for norovirus GII. Previously, we had shown that a reference-based mapping tool such as Metamaps [50] for long reads allowed us to separate closely related bacterial strains in the same food sample [46]. However, this tool did not give satisfactory result in this case study (data not shown) because of the shorter read length, low contamination dose, and low abundance of viral sequences in the associated database. A follow-up bioinformatics study might be able to find more specific tools to attain strain level for closely related strains of the same genogroup, at very low contamination level, in the same sample. However, the focus of this paper was to deliver a proof of concept at the wet-lab level and to obtain relatedness using a phylogenetic analysis, which is not possible with the conventional methods. Nevertheless, the analysis of the public dataset from Yang and colleagues allowed us to prove that our analysis workflow was performant for samples spiked with levels as low as 10^3^ genome copies. This improvement in detection level is probably due to the targeting of viral particles prior to the RNA extraction step. The sequencing technology presumably had no impact on the results, as fewer and shorter reads were produced with Illumina sequencing.

In conclusion, this study aimed at investigating which approach would be appropriate for further formal validation to be used for foodborne viral detection and full-genome based characterization. However, some further development would still be necessary before applying it in routine laboratories, as our results were not all positive and highlighted the complexity of such experiments when a virus is present at low dose in a sample. Some lessons learnt from our experiments with low contamination in food samples were that several methods that had been reported to give good results on higher contamination loads (e.g., clinical samples) did not work with our samples (i.e., poly(A) capture, rRNA depletion). Nevertheless, other methods we applied were able to characterize the norovirus spiked in food samples: notably, the shotgun metagenomics methods on cDNA (method C) or amplified cDNA method (method D) resulted in a consensus sequence covering 85% to 99% of the genome in the samples spiked at the highest concentration (10^7^ genome copies in 25 g of fruits). For a medium contamination dose (10^5^ genome copies in 25 g of fruits), a targeting approach such as SureSelect (method F) gave even better results, although it is very time-consuming, costly and does not allow for an open approach. Therefore, these methods that gave positive results in our study still have limitations. In the future, if improved, the adaptive sampling proposed by ONT could be a cheaper alternative that could also target more than one pathogen. For lower contamination doses, our developed bioinformatics workflow was able to detect and characterize norovirus and hepatitis A virus at doses as low as 10^3^ genome copies in 50 g of matrix, but with RNA extracted with another method after ultracentrifugation to enrich viral particles prior to extraction [18]. Although we initially thought that using the currently accredited RNA extraction protocol would be the easiest way to later implement this new approach in routine, as an alternative with access to an ultracentrifuge potentially not possible for all reference laboratories, and a pre-enrichment step being more time-consuming, we show in this study that there is a trade-off between straightforward applicability and the potential limit of detection. Notably, this limit of detection, i.e., the sensitivity, and the specificity and reproducibility of the method still have to be determined in follow-up validation studies, while this work only investigated the possibility to obtain whole genome characterization and phylogeny at a few different contamination loads as a proof of concept. These validations are not as common for metagenomics [51] as they are for qPCR tests, given the cost per sample. So far, there is no consensus on how to conduct these validations or how many samples are necessary [52], and in the case of norovirus, access to references materials spiked at various contamination levels will prove challenging as we are bound to the genome copies present in the spiked lenticules. Moreover, the costs and efforts of adapting the ISO-based routine sample preparation in reference laboratories should be carefully evaluated against the benefits obtained when using an improved RNA extraction protocol. Another limitation of this method, because it is based on nucleic acids, is the possible characterization of a pathogen in a non-infective state (not living). However, we believe that when a person was infected by ingesting contaminated food, it is important to find the source of the disease even if the pathogen is not infective anymore. Therefore, our focus was to obtain relatedness between cases using phylogenetic trees, for which a nucleic acid-based method proved successful. In addition, most of the metagenomics workflows including the one presented in this study still rely on the use of command lines and scripts, which is not straightforward for non-experts and prevents its use in routine settings, and should be addressed in the future. The databases that are used for bioinformatics analysis should also be continuously updated and completed, especially for the improvement of investigations of mixed datasets such as the metagenomics ones. It is, however, important to note that in all the cases where a characterization of the virus was possible, we were able to obtain a genome which could then be compared to other cases by phylogenetics, which goes well beyond the results obtained with the current methods of analysis of norovirus in food. This paper aimed at sharing some lessons learnt, including approaches that failed for our samples. We believe that this contribution is also important for the scientific community to grasp information on what should not be repeated in the future, and where to further focus community efforts. Above all, metagenomics is still a new approach and necessitates proofs of concept such as this one to advance the field, as was requested by EFSA [53].

## Figures and Tables

**Table 1:** Sequencing statistics for each of the sample preparation methods tested.

		Number of Sequenced Reads	Median Read Length	Median Read Quality	Read Length N50
shotgun cDNA Method C	munov1	27,406	471	9.8	634
	munov2	237,26	388	10.1	501
	hunov1	1,119,816	601	9.9	814
	hunov2	106,195	387	10.2	517
	Bk	10,143	900	9.4	1251
shotgun WTA method D	munov1	13,992,038	408	12.8	483
	munov2	2,576,015	345	12.8	421
	hunov1	15,151,030	405	13.2	609
	hunov2	22,591,785	401	12.6	542
	Bivalve	14,219,052	331	13	401
	Bk	3,898,276	310	13.2	351
WTA hybrid capture Method F	munov1	38,582,756	394	12.9	409
	munov2	28,497,819	431	18.2	494
	hunov1	12,473,896	328	12.8	338
	hunov2	14,357,765	391	12.8	407
	Bivalve	8,263,607	412	12.4	442
WTA Adaptive sampling method E	munov2_all_reads	1,535,739	333	12	368
	munov2_stop_receiving	161	453	12.6	505
	hunov2_all reads	3,269,856	370	12.9	404
	hunov2_stop_receiving	0	n/a	n/a	n/a
Flongle	munov1 wta (method D)	4810	476	7.6	573
	munov1 wta SS (method E)	12	294	3.3	4917
	hunov1 cDNA (method C)	6846	463	7.5	527
	hunov1 wta (method D)	10,119	363	7.6	416

munov = artificial spike of raspberries with murine norovirus at 10^7^ genome copies per 25 g (genogroup GV, RNA qPCR Cq: 26). hunov = artificial spike of raspberries with human norovirus GI at 10^5^ genome copies per 25 g (RNA qPCR Cq: 33). 1 and 2: biological replicates of the spiking. Bivalve: naturally contaminated bivalve sample qPCR-positive (RNA Cq: 34) for the presence of norovirus GII. Shotgun cDNA: reverse transcription of the extracted RNA (Method C). Shotgun WTA: cDNA after whole transcriptome amplification (Method D). WTA hybrid capture: cDNA after whole transcriptome amplification with target enrichment for norovirus using SureSelect (Method F). WTA adaptive sampling: cDNA after whole transcriptome amplification, sequenced with adaptive sampling (Method E). All reads: all reads sequenced during adaptive sampling. Stop-receiving: only reads matching to the database of norovirus and hepatitis A virus reference genomes during adaptive sampling. The results for methods A and B were not presented as no sequencing was conducted after negative qPCR result. n/a: not applicable.

**Table 2:** Results of data analysis of samples of raspberries spiked with murine norovirus (munov) in biological duplicates (munov1 and munov2), human norovirus (hunov) in biological duplicates (hunov1 and hunov2), a blank of raspberries (bk), and a naturally contaminated sample of bivalve positive for norovirus in qPCR (bivalve). Shotgun cDNA: complementary DNA after reverse transcription (method C). Shotgun WTA: cDNA after whole transcriptome amplification (Method D). WTA hybrid capture: cDNA after whole transcriptome amplification with target enrichment for norovirus using SureSelect (method F). WTA adaptive sampling: cDNA after whole transcriptome amplification, sequenced with adaptive sampling (Method E). All reads: all reads sequenced during adaptive sampling. Stop receiving: only reads matching to the database of norovirus and hepatitis A virus reference genomes during adaptive sampling. n/a: not applicable because norovirus not detected in the profiling step.

		Assembly				Data Analysis								
		Number of Contigs	Min Length	Max Length	N50	Kraken Unclassified %	Kraken Norovirus Contigs	Mash Best Norovirus Hit	Identity to the Reference (%)	Mash Matching Hashes	Coverage of the Reference (%)	BWA Number of Reads Mapping	Length Consensus Sequence	Genotype Consensus Sequence
**Shotgun cDNA** **Method C**	**munov1**	1098	256	3409	522	58	7	NC_008311.1	88	62/1000	85	35	6304	GV
	**munov2**	11,220	240	8,430	484	37	8	NC_008311.1	90	103/1000	99	155	7,280	GV
	**hunov1**	248,221	240	13,146	553	90	1	NC_031324.1	80	8/1000	16	86	1221	GI.3P3
	**hunov2**	9,750	241	12,527	578	81	0	n/a	n/a	n/a	n/a	n/a	n/a	n/a
	**Bk**	4088	269	7492	719	83	0	n/a	n/a	n/a	n/a	n/a	n/a	n/a
**Shotgun WTA** **method D**	**munov1**	55,942	240	3494	486	84	8	NC_008311.1	90	111/1000	91	743	6694	GV
	**munov2**	360,178	240	2576	421	50	11	NC_008311.1	91	147/1000	98	1312	7271	GV
	**hunov1**	458,249	240	4737	503	93	0	n/a	n/a	n/a	n/a	n/a	n/a	n/a
	**hunov2**	1,023,060	240	3692	443	89	0	n/a	n/a	n/a	n/a	n/a	n/a	n/a
	**Bivalve**	415,513	240	4842	437	93	0	n/a	n/a	n/a	n/a	n/a	n/a	n/a
	**Bk**	250,101	240	4482	443	94	0	n/a	n/a	n/a	n/a	n/a	n/a	n/a
**WTA hybrid capture** **Method F**	**munov1**	513,411	240	1681	378	84	6	NC_008311.1	91	135/1000	98	2786	7255	GV
	**munov2**	853,353	240	3448	372	36	16	NC_008311.1	90	183/1000	100	18,030	7381	GV
	**hunov1**	144,897	240	4682	349	91	2	NC_031324.1	85	32/100	40	301	3115	GI
	**hunov2**	328,106	240	1671	370	70	18	NC_031324.1	89	80/1000	78	2636	6071	GI
	**Bivalve**	149,962	240	1614	377	63	0	n/a	n/a	n/a	n/a	n/a	n/a	n/a
**WTA adaptive sampling** **method E**	**munov2_all_reads**	210,832	240	2538	403	54	7	NC_008311.1	91	124/1000	95	711	6978	GV
	**munov2_stop_receiving**	15	299	1006	498	33	9	NC_008311.1	90	104/1000	61	161	4474	GV
	**hunov2_all reads**	358,200	240	2148	443	92	0	n/a	n/a	n/a	n/a	n/a	n/a	n/a
	**hunov2_stop_receiving**	n/a	n/a	n/a	n/a	n/a	n/a	n/a	n/a	n/a	n/a	n/a	n/a	n/a
**Flongle**	**munov1 wta**	849	300	1452	541	89	0	n/a	n/a	n/a				
	**hunov1 cDNA**	1383	269	2257	538	82	0	n/a	n/a	n/a				
	**hunov1 wta**	1708	300	1,680	433	92	0	n/a	n/a	n/a				

**Table 3:** Results of data analysis of samples of celery spiked with norovirus (NOV) and/or HAV [18]. -1: first biological replicate. -2: second biological replicate. When two viruses were spiked, the best mash hit of each species was presented along with the result of the mapping and typing for each strain. n/a: not applicable (analysis was not continued because only 1 strain detected when 2 strains were spiked). Sequence read lengths: 35–100 bp.

			Assembly				Taxonomic Classification			Data Analysis						
Accession Number	Sample Description (Spike Description)	Number of Sequenced Reads	Number of Contigs	Min Length	Max Length	N50	Kraken Unclassified %	Number of Contigs Norovirus	Number of Contigs HAV	Mash Best Norovirus Hit	Identity to the Reference (%)	Mash Matching Hashes	Coverage of the Reference (%)	BWA Number of Reads Mapping	Length Consensus Sequence	Genotype Consensus Sequence
SRR5352286	NOV 10^5^	2,090,232	120	202	7680	783	24	1	0	NC_029646.1 (NOV)	86	40/1000	55	11,104	4137	GII
SRR5353140	NOV 10^4^ -1	2,056,188	163	201	4233	611	40	2	0	NC_029646.1 (NOV)	86	43/1000	55	15,587	4142	GII
SRR5353141	NOV ^104^ -2	2,478,355	113	200	7578	898	4	1	0	NC_029646.1 (NOV)	85	37/1000	56	16,649	4209	GII.P4
SRR5353142	NOV 10^3^ -1	1,879,486	1296	200	4546	509	85	3	0	NC_029646.1 (NOV)	83	19/1000	30	1416	2,23	GII
SRR5353143	NOV 10^3^ -2	2,072,984	1903	200	3576	503	88	2	0	NC_029646.1 (NOV)	84	26/1000	35	1666	2618	GII.P4
SRR5353144	NOV GII.4 10^6^ + NOV GII.6 10^1^ -1	2,335,180	14,741	200	7444	692	94	3	0	NC_029646.1 (NOV)	84	25/1000	n/a	n/a	n/a	n/a
SRR5353145	NOV GII.4 10^6^ + NOV GII.6 10^1^ -2	2,546,316	13,276	200	7176	642	94	3	0	NC_029646.1 (NOV)	83	19/1000	n/a	n/a	n/a	n/a
	NOV GII.4 10^3^ + NOV GII.6 10^4^ -1	2,705,565	15,329	200	7076	711	94	4	0	NC_029646.1 (NOV)	72	1/1000	n/a	n/a	n/a	n/a
SRR5353159	NOV GII.4 10^3^ + NOV GII.6 10^4^ -2	2,203,937	13,245	200	7076	665	94	7	0	NC_029646.1 (NOV)	72	1/1000	n/a	n/a	n/a	n/a
SRR5353212	NOV 10^6^ + HAV 10^6^ -1	2,109,188	9122	201	7471	605	94	4	1	NC_029646.1 (NOV) NC_001489.1 (HAV)	84 99	29/1000 898/1000	41 99	3554 6524	3069 7462	GII.P4 HAV I.B
SRR5353213	NOV 10^6^ + HAV 10^6^ -2	1,736,984	7706	201	7471	606	93	1	1	NC_029646.1 (NOV) NC_001489.1 (HAV)	82 99	14/1000 896/1000	23 99	1713 5484	1708 7460	GII.P4 HAV I.B
SRR5353214	NOV 10^4^ + HAV 10^7^ -1	221,046	674	201	5684	442	89	0	2	NC_001489.1 (HAV)	99	889/1000	99	3383	7438	HAV I.B
SRR5353215	NOV 10^4^ + HAV 10^7^ -2	797,912	4416	201	5803	538	93	0	2	NC_001489.1 (HAV)	99	894/1000	100	5614	7468	HAV I.B

## Data Availability

All data are publicly available under BioProject PRJNA878666.

## Supplementary Materials

The following supporting information can be downloaded at: https://www.mdpi.com/article/10.3390/foods11213348/s1, Table S1: list of norovirus and hepatitis A virus reference genomes used for adaptive sampling.
